# DNA Hypomethylation Is Not Cell Intrinsically Toxic to Polycomb Repressive Complex 2 Deficient Malignant Peripheral Nerve Sheath Tumors

**DOI:** 10.1002/gcc.70156

**Published:** 2026-08-04

**Authors:** Madilyn R. Stahl, Christopher M. Stehn, Zachary J. Seeman, Christopher Faulk, David A. Largaespada

**Affiliations:** ^1^ Department of Pediatrics, Masonic Cancer Center University of Minnesota Minneapolis Minnesota USA; ^2^ Department of Genetics, Cell Biology and Development University of Minnesota, Twin Cities Minneapolis Minnesota USA; ^3^ Department of Animal Science, College of Food, Agricultural and Natural Resource Sciences University of Minnesota Minneapolis Minnesota USA

**Keywords:** DNA methylation, DNMTi, MPNST, NF1, PRC2

## Abstract

Malignant peripheral nerve sheath tumors (MPNSTs) are aggressive soft tissue sarcomas and the most common cause of disease‐associated death for neurofibromatosis type 1 (NF1) patients. In the context of NF1, MPNSTs develop from benign premalignant precursors and the transition to malignancy is typically accompanied by loss of the polycomb repressive complex 2 (PRC2), which results in aberrant upregulation of over 1200 genes due to global depletion of histone H3 lysine 27 trimethylation (H3K27me3). Previous studies suggest cells compensate for the loss of this repressive histone mark via hypermethylation of the genome. Here we analyzed genome‐wide DNA methylation and the transcriptome in MPNST cell lines and isogenic PRC2‐deficient and ‐proficient CRISPR‐engineered immortalized human Schwann cells. In addition to effects of PRC2 status, we also measured the effects of two DNA methyltransferase inhibitors (DNMTi), decitabine and azacitidine. We found that PRC2 status does not affect global DNA methylation or average methylation levels across specific genomic features. Furthermore, decitabine and azacitidine have differential effects on MPNSTs. While both DNMTis hypomethylate the genome, they upregulate different targets. Azacitidine upregulates genes involved in RNA processing pathways and exhibits direct tumor cell cytotoxicity, while decitabine upregulates genes involved in the immune response, has no direct‐cell killing effects, and likely suppresses tumor growth in vivo by altering the tumor microenvironment. We show that DNA hypomethylation alone is insufficient to kill MPNST cells, regardless of PRC2 status. Consequently, these findings suggest that DNMT inhibitors should be utilized in combination with other targeted therapies for MPNST patients.

## 1 Introduction

Malignant peripheral nerve sheath tumors (MPNSTs) are highly aggressive sarcomas that are rare in the general population but represent the most common cause of disease‐associated death in patients with neurofibromatosis type 1 (NF1) syndrome [1]. NF1 is a cancer predisposition syndrome that arises from the inheritance of one loss‐of‐function copy of the *NF1* gene, which encodes neurofibromin, a RAS GTPase‐activating protein (GAP). Individuals with NF1 syndrome are predisposed to the development of both benign and malignant tumors, including malignant peripheral nerve sheath tumors (MPNSTs). MPNSTs are composed of neoplastic Schwann lineage cells and arise through a well‐characterized genetic pathway [2]. First, the remaining wild type *NF1* allele is lost, which results in biallelic loss of *NF1* and neurofibromin function. This loss, taken together with non‐genomic changes, including changes to the tumor microenvironment, causes the development of plexiform neurofibromas (PN) in deep peripheral nerve sheaths [3]. Next, loss‐of‐function mutations or deletions of *CDKN2A*, often together with the loss of *CDKN2B*, result in the development of an atypical neurofibromatous neoplasm of unknown biological potential (ANNUBP), the precursor to MPNST [4, 5]. The development of MPNST is accompanied by chromosome instability, tetraploidy, and genome‐wide copy number alterations, but is driven by biallelic loss‐of‐function mutations in either *SUZ12* or *EED* in 80% of cases [6, 7, 8]. *SUZ12* and *EED* protein products form the core component of Polycomb repressive complex 2 (PRC2), which catalyzes the mono‐, di‐, and tri‐methylation of lysine 27 on histone H3, and silences gene expression to maintain cell identity [8, 9, 10]. Accordingly, MPNSTs with PRC2 inactivation show near complete loss of histone H3 lysine 27 trimethylation (H3K27me3). In the absence of PRC2‐mediated repression, MPNSTs show increased histone H3 lysine 27 acetylation (H3K27ac), a mark of active transcription associated with aberrant, ectopic gene expression of over 1200 genes [8, 11].

The relationship between PRC2 loss and DNA methylation in MPNST remains a subject of debate. Some studies have reported slight hypomethylation or no global change in DNA methylation levels in PRC2‐deficient MPNST while other studies have shown PRC2 loss induces genome‐wide hypermethylation, potentially as a compensatory response to the loss of repression by PRC2 [12, 13, 14, 15]. The hypothesis of compensatory hypermethylation suggests that PRC2‐deficient cells become uniquely dependent on DNA methyltransferases (DNMT) to maintain gene silencing [12]. Consequently, this shift is thought to cause a therapeutic vulnerability that can be exploited using DNMT inhibitors (DNMTi). In PRC2‐deficient MPNST cells, DNMTi have been shown to induce viral mimicry and cause cell death through the activation of endogenous retroviruses, which form dsRNA and trigger the interferon response [13, 16, 17].

Two DNMTis, decitabine and azacitidine, are currently FDA‐approved for the treatment of acute myeloid leukemia and myelodysplastic syndrome [18]. Inside the cell, decitabine is converted into an active triphosphate which incorporates into DNA and irreversibly binds DNMTs, thereby preventing DNA methylation during replication [19]. In contrast, only about 15% of azacitidine is reduced into this same DNA‐incorporating triphosphate form to perform its DNMTi function, while the remaining majority is converted into a ribo‐triphosphate that is incorporated into RNA, disrupting RNA processing and protein synthesis [20, 21, 22]. The suitability of DNMTi for MPNST treatment, and the potential of PRC2 status as a biomarker of sensitivity, are important issues because decitabine is currently being investigated under a phase II clinical trial in patients with PRC2‐deficient MPNST (NCT04872543).

To evaluate the efficacy of DNMTi in PRC2‐deficient MPNST, we first established baseline global DNA methylation across our cell line panel using Nanopore genome skimming. Genome skimming is a low‐pass sequencing method and typically used in evolutionary studies to analyze high‐copy regions, such as ribosomal DNA and the mitochondrial genome [23, 24, 25]. Recently, it has been used to calculate methylation fractions of the whole genome and repeat element families due to the direct 5mC sequencing nature of Nanopore [26]. Following the characterization of baseline global DNA methylation, we assessed the potency of both inhibitors in inducing cell death and apoptosis in vitro and tumor control in an in vivo xenograft model. To elucidate the underlying mechanisms, we then quantified DNMTi‐induced hypomethylation, quantified changes in gene expression, and identified differentially regulated pathways.

Our results demonstrate that PRC2 status does not significantly alter baseline global DNA methylation levels or the average methylation of specific genomic features in MPNST cells. Furthermore, we show that while decitabine and azacitidine both effectively reduce DNA methylation, they operate through distinct but overlapping molecular mechanisms and therefore regulate distinct gene sets. Notably, our findings indicate that DNMTi‐induced hypomethylation is not intrinsically toxic to MPNST cells, suggesting that PRC2 deficiency does not sensitize these tumors to global methylation loss.

## 2 Materials and Methods

### 2.1 Tissue Culture

MPNST cell lines S462TY, ST88‐3, ST88‐14, STS26T, MPNST‐724, and NF90.8 were kindly provided by Dr. Nancy Ratner (Cincinnati Children's Hospital). JH‐2‐002 CL was provided by Dr. Christine Pratilas (Johns Hopkins), MPNST‐642 was received from the Characterized Cell Line Core Facility (MD Anderson Cancer Center), and sNF96.2 was purchased from American Type Culture Collection (ATCC). Immortalized human Schwann cells (iHSCs) were made in our lab [11, 27]. We used three “addback” models, where PRC2 function was restored via re‐expression of *SUZ12*. S462TY was sequentially transduced with vectors containing a reverse tetracycline‐controlled transactivator (rtTA) cDNA and a tetracycline‐response element (TRE) promoter‐driven SUZ12 cDNA, respectively. Following each transduction, selection was done with hygromycin at 500 ng/mL and puromycin at 5 ng/mL. Post‐selection, single‐cell clones were selected to obtain populations for testing restoration of H3K27me3, with optimal restoration observed under treatment of doxycycline at 500 ng/mL for 7 days. The ST88‐14 and JH‐2‐002 CL addbacks were kindly provided by Dr. Jack Shern (National Institutes of Health) and treated with doxycycline at 500 ng/mL for 5 days [28]. Media with doxycycline was replenished every 2 days. All cell lines were maintained at 37°C with 5% CO_2_ and were routinely tested for mycoplasma. Cell lines and media are listed in Table S1.

### 2.2 In Vitro Drug Testing

Drug viability testing on azacitidine and decitabine (SelleckChem) was performed as previously described, with minor modifications as described below [29]. Cells were plated into 384‐well plates with a Biomek 2000 at 1000 cells per well. Following a 24‐h incubation, the drug was added using an Echo 550 liquid dispenser in quadruplicate in a 12‐point dose–response manner. Cell viability was assessed with alamarBlue after 96 h of continuous incubation with the drug, and fluorescence was read on a CLARIOstar microplate reader. Dose response curves were generated in Prism using the nonlinear regression log(inhibitor) versus response‐variable slope model.

### 2.3 Animal Testing

All in vivo procedures were approved by the Institutional Animal Care and Use Committee (IACUC) at the University of Minnesota. For the generation of tumors, 1.0 × 10^6^ S462‐TY cells in 50% Matrigel (BD Biosciences) were injected subcutaneously into the flank of 5‐week‐old NOD‐Rag1null IL2rgnull, NOD rag gamma, NOD‐RG (NRG) mice. Equal numbers of males and females were used. Animals were randomized to treatment (*n* = 5 per arm) or control arms (*n* = 4 per arm). Drugs were administered via intraperitoneal (IP) injection. Treatment began once tumors reached 500 mm^3^ and was continued for 2 weeks. Azacitidine (SelleckChem) was resuspended in 5% DMSO, 30% PEG400, and 65% H_2_O and was treated at 5 mg/kg daily [30, 31]. Decitabine (SelleckChem) was resuspended in PBS and was treated at 5 mg/kg three times a week [13, 32].

### 2.4 Genome Skimming

Cells were treated with azacitidine, decitabine, or DMSO for 96 h, with a media change and drug replenishment after 48 h of incubation. DNA was extracted from cells using the GeneJET Genomic DNA Purification Kit (Thermo Scientific). Extracted DNA was quantified using a Qubit Fluorometer (Invitrogen). Libraries were prepped using the Native Barcoding Kit 96 V14 (Oxford Nanopore) and sequenced on a PromethION 2 Solo. Data analysis was performed as previously described on the Minnesota Supercomputing Institute's HPC cluster, aligning reads to the genome assembly T2T‐CHM13v2.0 [26]. To evaluate the effect of PRC2 status on DNA methylation, technical triplicates were averaged for each cell line and compared using a *t*‐test in R.

### 2.5 RNA‐Seq

RNA was extracted from cells treated with azacitidine, decitabine, or DMSO for 96 h, with a media change and drug replenishment after 48 h, using the RNEasy mini purification kit (Qiagen). TURBO DNase (Invitrogen) was added to remove genomic DNA contamination. RNA was submitted to the University of Minnesota Genomics Center for quality assessment with the Aligent Bioanalyzer, for library preparation with the TruSeq Stranded mRNA kit, and for sequencing with 150 bp paired end reads for 30 million reads per sample on an Illumina NovaSeq X+ or an Element Biosciences AVITI. Data were processed with the CHURP pipeline on the Minnesota Supercomputing Institute's HPC cluster [33]. In R, differential gene expression analysis was performed using DESeq2 [34]. Count data were transformed using the regularized log (rlog) transformation, and inclusion criteria for differentially expressed genes were an absolute fold change ≥ 1 with a false discovery rate (FDR) < 0.05. GSEA was used for pathway analysis with the top 500 differentially expressed genes [34, 35, 36, 37]. Pathways with an adjusted *p* < 0.1 were considered significant. TETranscripts was used for the analysis of repeat element expression [38].

### 2.6 Immunohistochemistry

Tissue samples were formalin fixed, paraffin embedded, and cut into 5 μm sections. Sections were then deparaffinized and hydrated. Antigen retrieval was performed using citrate buffer. Endogenous peroxidases were blocked with H_2_O_2_, and nonspecific binding was blocked overnight at 4°C with 5% goat serum in TBS‐T. Sections were incubated with Cell Signaling Technology primary antibodies diluted in 5% goat serum in TBS‐T overnight at 4°C (CD11b #77699, 1:100; F4/80 #70076, 1:200; CD31 #77699, 1:100). After washing, sections were treated with biotinylated goat anti‐rabbit antibody for 1 h. Sections were washed, treated with VECTASTAIN Elite ABC‐HRP (Vector), washed, and stained with ImmPACT DAB Substrate (Vector). Sections were stained with hematoxylin, dehydrated, and mounted in permount. Slides were imaged with a Cytation 5 (Aligent), and images analyzed in Fiji [39].

### 2.7 Flow Cytometry

CytoFLEX S and FlowJo v10 were used for flow cytometry analysis on cells treated with azacitidine, decitabine, or DMSO control for 24, 48, and 96 h, with drug replenished after 48 h. Etoposide was used as a positive control for apoptotic cells treated for 24 h at 20 μM. Single cell suspensions were stained with Caspase‐3/‐7 (Invitrogen #V35118) and Annexin V (Invitrogen #A35122) according to manufacturer protocols.

## 3 Results

### 3.1 Global DNA Methylation Levels Are Not an Indicator of PRC2 Status

To determine whether global DNA methylation levels differ with PRC2 loss, we used genome skimming to measure global DNA methylation levels in PRC2‐deficient and PRC2‐proficient MPNST cell lines. We analyzed several established MPNST cell lines, *NF1*‐deficient iHSCs with either *SUZ12* or *EED* knockouts to model PRC2 loss, and MPNST “addback” cell lines (S462TY‐500‐5, JH‐2‐002 CL, and ST88‐14) in which PRC2 function was restored through doxycycline‐inducible tet‐regulated SUZ12 expression (Figure S1) [11, 27, 28]. All samples were sequenced in triplicate at a minimum of 0.05X coverage, which is sufficient to accurately estimate the global DNA methylation fraction [26].

**FIGURE 1:**
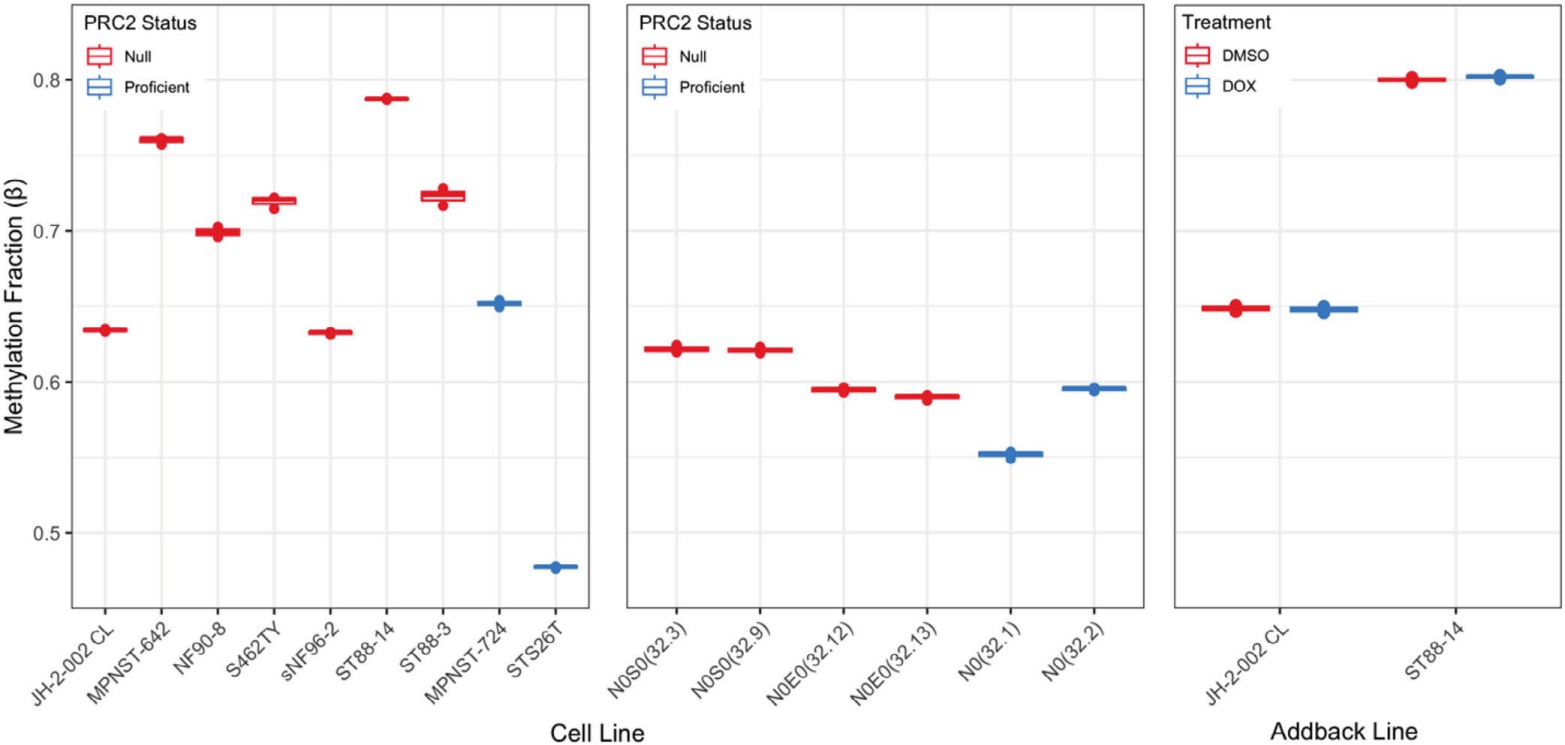
Global DNA methylation fraction (*β*) of MPNST cell lines, iHSCs, and addback cell lines. Color indicates PRC2 status.

Global DNA methylation levels were not significantly altered by PRC2 status across any of the models tested (Figure 1). Average DNA methylation fractions of MPNST cell lines were not significantly different (*p* = 0.335) between PRC2‐proficient (0.56 ± 0.10, *n* = 2 cell lines) and PRC2‐deficient samples (0.71 ± 0.06, *n* = 7 cell lines). Similarly, no significant differences (*p* = 0.343) were observed in the isogenic iHSCs in which PRC2‐proficient samples averaged 0.57 ± 0.02 (*n* = 2 cell lines) and PRC2‐deficient samples averaged 0.61 ± 0.02 (*n* = 4 cell lines). Finally, in MPNST SUZ12 addback lines, methylation levels were nearly identical (*p* = 0.996) between PRC2‐proficient (0.72 ± 0.08, *n* = 2 cell lines) and PRC2‐deficient samples (0.73 ± 0.08, *n* = 2 cell lines). In conclusion, these results show that PRC2 status does not affect global DNA methylation levels across three MPNST models, including two isogenic systems.

Though genome skimming is not sufficient to investigate locus‐specific changes, we determined average methylation levels in the iHSCs at all transcription start sites (TSS), CpG islands (CGIs), Alu elements, LINE1 elements, and human endogenous retroviruses (hERVs) (Figure S2). We identified no significant changes in DNA methylation in any of these sites between PRC2‐proficient (two cell lines) and PRC2‐deficient (four cell lines) samples. However, these data do not exclude the site‐specific changes that could occur with PRC2 loss.

### 3.2 Decitabine‐Induced DNA Hypomethylation Is Not Intrinsically Toxic to MPNSTs Regardless of PRC2 Status

**FIGURE 2:**
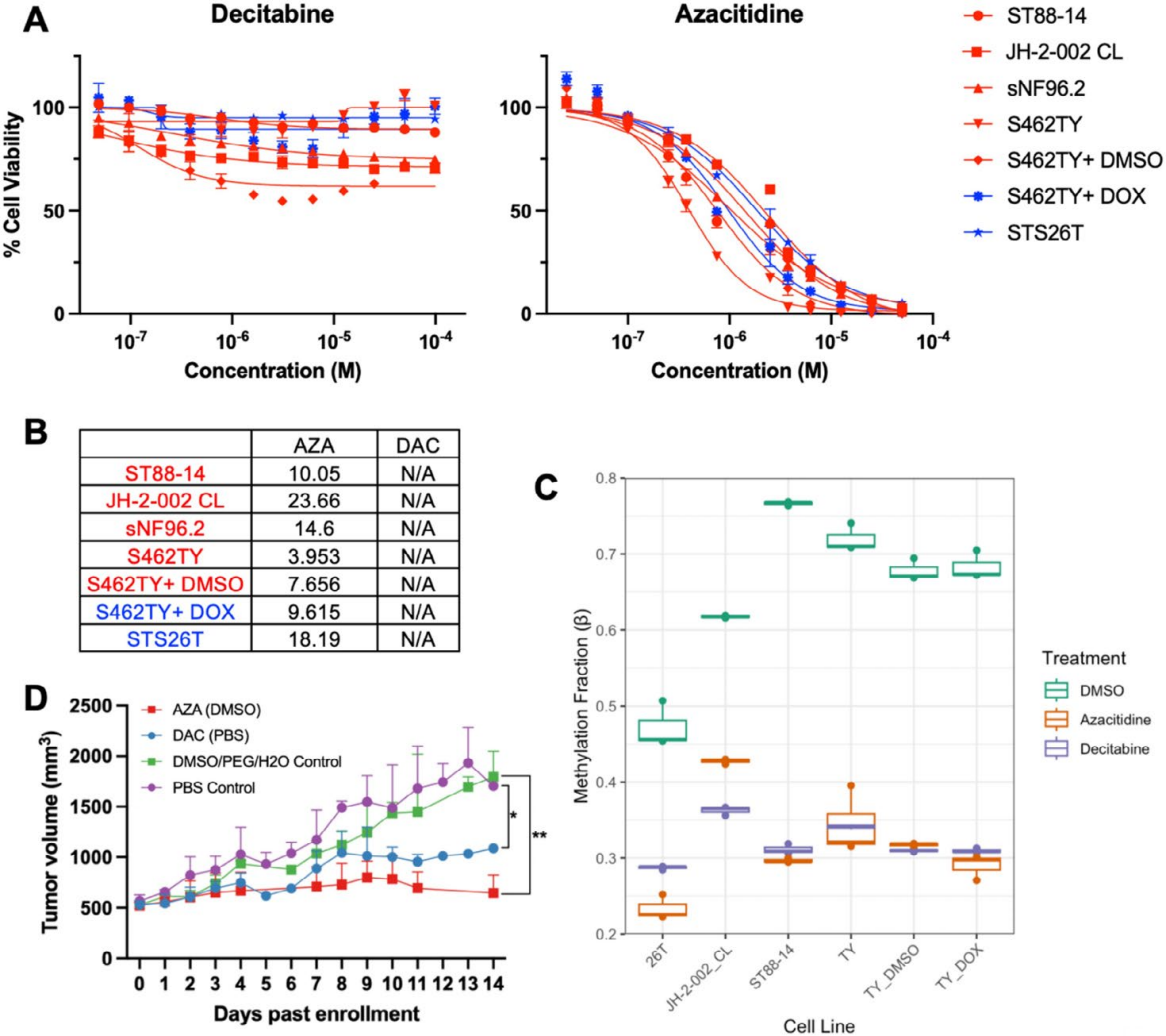
Differential killing of decitabine and azacitidine. (A) Dose response curves of decitabine (left) and azacitidine (right) in several MPNST cell lines. Red indicates PRC2 deficient, blue indicates PRC2 proficient. (B) Average half maximal inhibitory concentration (IC_50_) of azacitidine and decitabine in μM. (C) Genome‐wide methylation fraction of DNMTi‐treated MPNST cell lines. Cell lines labeled “DMSO” and “DOX” are addbacks, where doxycycline induces the expression of *SUZ12* to restore PRC2 function. (D) Tumor growth curves for mice that received azacitidine (*p* = 0.003), decitabine (*p* = 0.01), or vehicle control.

DNMTis have been proposed as a possible therapeutic for MPNSTs and are currently being studied in a clinical trial, with PRC2 loss suggested as a biomarker of sensitivity [13]. However, we found that decitabine does not kill MPNST cell lines at concentrations up to 1000 μM. On the other hand, azacitidine effectively kills MPNSTs in cell lines with an average IC_50_ of 12.5 μM (Figure 2A,B). Consistent with this, only azacitidine induced apoptosis in MPNST cell lines (Figure S3). To determine whether these effects were associated with changes in global DNA methylation, we performed Nanopore genome skimming. Azacitidine was administered at its IC_50_ for each cell line, whereas decitabine was administered at 0.15 μM to produce a comparable level of overall demethylation. As expected, we found that both DNMTis greatly decreased global DNA methylation (Figure 2C). These findings demonstrate that although both agents effectively reduce global DNA methylation, only azacitidine induces apoptosis, indicating that hypomethylation alone does not account for therapeutic efficacy in this context.

In contrast to our in vitro data, both azacitidine and decitabine significantly slowed tumor growth in mice with MPNST cell line xenografts compared to vehicle controls (Figure 2D). Although the xenograft studies were performed in immunodeficient mice, myeloid cells remain intact. Therefore, we assessed immune cell infiltration in the cell lines via IHC (Figure S4). While total immune cell infiltration was unchanged, we did observe a decrease in F4/80, a macrophage marker, in decitabine‐treated tumors. Additionally, we observed reduced CD31 in decitabine‐treated tumors, revealing reduced angiogenesis. We observed no significant differences in immune cell infiltration or angiogenesis in azacitidine‐treated tumors compared to vehicle controls. This suggests that the differences observed between our in vitro and in vivo studies may be explained by the effects of decitabine on the tumor microenvironment.

### 3.3 Azacitidine and Decitabine Have Differential Effects on Gene Expression

**FIGURE 3:**
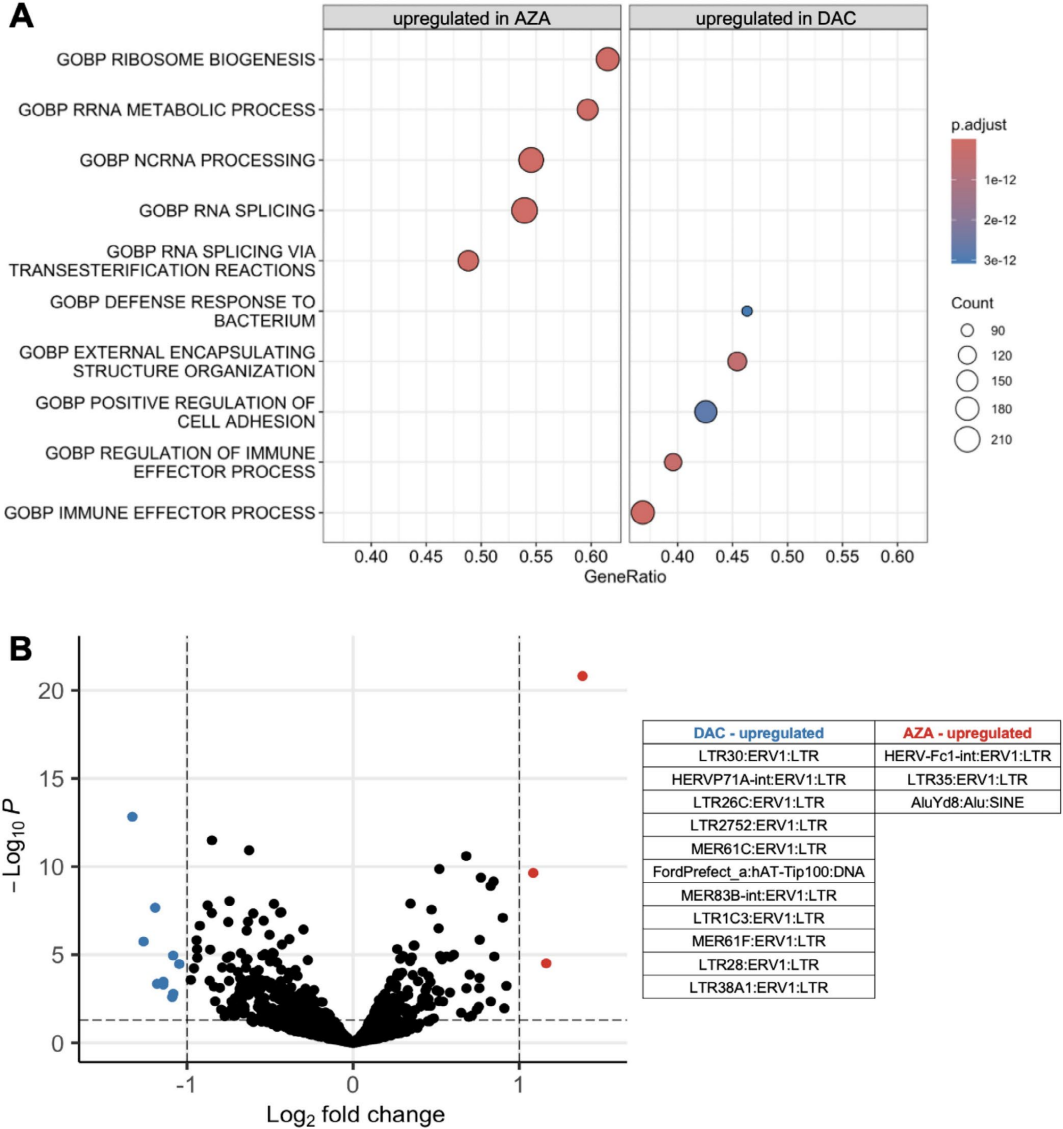
Azacitidine and decitabine induce unique transcriptional responses. (A) Gene Ontology Biological Pathways upregulated in MPNST cell lines by azacitidine (left) and decitabine (right). (B) Transposable element family expression upregulated by azacitidine (red) and decitabine (blue).

To investigate the molecular basis for the observed differences in cell killing of the two DNMTi with approximately the same amount of DNA hypomethylation, we characterized the transcriptomic changes induced by each DNMTi in MPNST cells. Through pathway analysis of RNA‐Seq data, we found that the top five Gene Ontology Biological Processes upregulated in azacitidine when compared to decitabine are all involved in RNA processing (Figure 3A). In contrast, decitabine upregulates genes involved in immune response signatures more potently than azacitidine (Figure 3A). Though both drugs function as DNMTi to reduce DNA methylation and increase the expression of genes compared to DMSO control (Figure S5), we hypothesize that the inhibition of RNA processing by azacitidine is more important for killing MPNST cells directly.

Previous reports have suggested that decitabine induces a viral mimicry response to kill cells, so we utilized RNA‐Seq and an R package, TEtranscripts, to quantify transposable element (TE) expression [38]. We observed that decitabine derepresses a broader repertoire of TE families than azacitidine, with long terminal repeat (LTR) retroelements being the most significantly upregulated family across both treatments (Figure 3B). These data suggest that the specific genes and TEs induced by decitabine are enriched for immune‐related pathways. This provides a mechanistic basis for our observation that decitabine, while not cytotoxic in vitro, facilitates tumor control in vivo, perhaps by stimulating a T cell‐independent immune response.

## 4 Conclusions

DNA methylation has been proposed as a critical compensatory repressive mark in PRC2‐deficient MPNST, and based on this rationale, a clinical trial using the DNMTi decitabine was initiated for these tumors [12, 13]. However, we observed no significant differences in global DNA methylation or in average methylation levels across specific genomic features (TSS, CGIs, Alu elements, LINE1 elements, hERVs) associated with PRC2 status in three in vitro models of MPNST. While genome skimming allowed us to assess average methylation levels across key genomic features, this approach is not sufficient to capture locus‐specific alterations. Therefore, site‐specific epigenetic rewiring, rather than global DNA methylation changes, may drive the biology of these PRC2‐deficient tumors. We tested decitabine and azacitidine in our models and confirmed that both greatly hypomethylate the genome. However, we observed no cell killing or apoptosis‐inducing effects of decitabine in vitro. Azacitidine, of which approximately 15% is metabolized into the active form of decitabine, effectively killed MPNST cell lines and induced apoptosis. The primary function of azacitidine is to incorporate into RNA, to inhibit RNA processing and protein synthesis [21, 22]. Together, these data suggest that hypomethylation alone is insufficient to drive cell death in MPNST, and the inhibition of RNA processing and protein synthesis via azacitidine is more important for direct cell killing.

Here, we show that decitabine is less effective than azacitidine at killing MPNST cells in vitro and in vivo. We hypothesize that the slowed tumor growth observed with decitabine treatment is most likely due to its effects on angiogenesis and immune cell infiltration, and not direct effects on the tumor cells. We observed a significant reduction in CD31, an angiogenesis marker, and F4/80, a macrophage marker, in decitabine‐treated tumors, but not azacitidine‐treated tumors. This data agrees with a previous report which linked the reduction of angiogenesis via decitabine to the reduction of angiogenic VEGF variants and an increase in an anti‐angiogenic variant [40]. Furthermore, there have been multiple reports showing reduction in angiogenesis leads to an inhibition of MPNST growth in vivo [41, 42]. The decrease in macrophages we observed in decitabine‐related tumors is likely tumor‐associated macrophages (TAMs), which are implicated in the development and progression of many cancers, including MPNST [43, 44, 45]. Lastly, we found that decitabine upregulates a broader range of TE families than azacitidine, which may promote viral mimicry and stimulate immune activation [16, 17]. Though we could not test the role of a T or NK cell immune response in our NRG mouse model as they lack these cells, we hypothesize that a stronger viral mimicry‐induced immune response would occur in an immunocompetent model with decitabine. To conclude, we hypothesize that the reduction of tumor growth by decitabine may be caused by indirect effects on the tumor microenvironment while azacitidine directly kills MPNST cells.

In contrast to the findings reported by Wojcik et al. [12], we do not observe an impact of PRC2 status on global DNA methylation or on cellular sensitivity to the hypomethylating agent decitabine in our models. While Wojcik et al. [12] and more recently Patel et al. [13], described decitabine sensitivity in their context, our data from multiple isogenic human MPNST and immortalized Schwann cell systems with defined PRC2 loss or restoration show no consistent differences in global methylation landscapes and no enhanced susceptibility to decitabine upon PRC2 disruption. The use of well‐controlled isogenic systems minimizes confounding genetic background effects, strengthening our confidence that PRC2 loss alone is not sufficient to alter methylation or confer decitabine sensitivity in MPNST.

In conclusion, our data highlight distinct therapeutic profiles for two hypomethylating agents in MPNST. While decitabine appears to suppress tumor growth in vivo, likely by altering the tumor microenvironment, azacitidine demonstrates superior direct tumor cell cytotoxicity in vitro. Given its robust capacity for direct cell killing, azacitidine remains a highly promising clinical candidate, though further in vivo studies in immunocompetent models are warranted to definitively establish its therapeutic superiority. Notably, the effects of both agents do not appear specific to PRC2‐deficient tumors. Additionally, we found no significant differences in global DNA methylation associated with PRC2 status across iHSCs, MPNST cell lines, or PRC2 addback models. We also observed no significant differences in average methylation of genomic features associated with PRC2 status in our immortalized human Schwann cell model. Ultimately, our work highlights that genomic hypomethylation alone is insufficient to induce cell death in MPNST, suggesting that DNMT inhibitors should be utilized in combination with other targeted therapies for MPNST patients.

## Author Contributions

**Madilyn R. Stahl** and **David A. Largaespada:** designed the study. **Christopher Faulk** and **David A. Largaespada:** advised the study. **David A. Largaespada:** acquired funding. **Madilyn R. Stahl** and **Christopher M. Stehn:** acquired and generated cell lines. **Madilyn R. Stahl** and **Zachary J. Seeman:** performed experiments. **Madilyn R. Stahl:** analyzed data and performed bioinformatic analysis and wrote the manuscript.

## Funding

This work was supported by the National Institutes of Health (CA151845 and CA154998 to D.A.L. and P30 CA77598), American Cancer Society Research Professor Award (RP‐17‐216‐06‐COUN to D.A.L.), Zachary NF Research Fund, and Children's Cancer Research Fund.

## Conflicts of Interest

D.A.L. is the co‐founder and co‐owner of NeoClone Biotechnologies Inc., Discovery Genomics Inc. (recently acquired by Immusoft Inc.), B‐MoGen Biotechnologies Inc. (recently acquired by Bio‐Techne Corporation), and Luminary Therapeutics Inc. He also owns equity in and serves as a scientific advisor to Styx Biotechnologies. The business of all these companies is unrelated to the contents of this manuscript. The other authors declare no conflicts of interest.

## Supporting information


**Table S1:** Media used for each cell line. iHSCs include N0S0(32.3), N0S0(32.9), N0E0(32.12), N0E0(32.13), N0(32.1), and N0(32.2). Addback cell lines used the same media as their parental cell line +/− doxycycline.
**Figure S1:** PRC2 restoration in S462‐TY “addback” cell line. Western blot showing restoration of SUZ12 and H3K27me3 with treatment of doxycycline at 500 ng/mL for 7 days.
**Figure S2:** PRC2 status does not alter DNA methylation patterns across global genomic features in iHSCs. Average DNA methylation percentages quantified across specific genomic features. Data show representative replicates for each cell line (*n* = 2 PRC2‐proficient cell lines, blue; *n* = 4 PRC2‐deficient cell lines, magenta).
**Figure S3:** Differential apoptosis induction of decitabine and azacitidine. Percent of live and apoptotic cells via flow cytometry of Annexin V and Caspase 3/7 on cells treated with decitabine, azacitidine, and DMSO control (*N* = 3). Cells were treated with azacitidine at its IC50 for each cell line, decitabine was treated at 0.15 μM to achieve approximately the same level of demethylation, and etoposide was treated at 20 μM for 24 h.
**Figure S4:** Decitabine reduces angiogenesis and macrophage infiltration. Staining of CD11b, F4/80, and CD31 in tumors treated with azacitidine, decitabine, or vehicle control. Dotted line is average % tissue stained in secondary only control. Scale bar 200 μm.
**Figure S5:** Azacitidine and decitabine induce the expression of many genes. Volcano plots showing many upregulated genes in both azacitidine (left) and decitabine (right) when compared to DMSO control.

## Data Availability

The data that support the findings of this study are openly available in SRA at https://dataview.ncbi.nlm.nih.gov/object/PRJNA1298274 (Nanopore genome skimming) and GEO at https://www.ncbi.nlm.nih.gov/geo/query/acc.cgi?acc=GSE305426 (RNA‐seq).
