# Supplementary material for: DNA Hypomethylation Is Not Cell Intrinsically Toxic to Polycomb Repressive Complex 2 Deficient Malignant Peripheral Nerve Sheath Tumors
Source: Genes Chromosomes Cancer. 2026 Aug 4;65(8):e70156. doi: 10.1002/gcc.70156 (PMC13437926; doi:10.1002/gcc.70156)
Supplement: Supplementary file 1 [file GCC-65-e70156-s001.docx]

**SUPPLEMENTARY DATA**

**Supplementary Table S1. Media used for each cell line.** iHSCs include N0S0(32.3), N0S0(32.9), N0E0(32.12), N0E0(32.13), N0(32.1), and N0(32.2). Addback cell lines used the same media as their parental cell line +/- doxycycline.

| **Cell line** | **Media** |
| --- | --- |
| S462TY | DMEM + 10% FBS + P/S |
| ST88-3 | DMEM + 10% FBS + 2 mM L-glutamine + 1 mM sodium pyruvate + P/S |
| ST88-14 | DMEM + 10% FBS + P/S |
| STS26T | DMEM + 10% FBS + P/S |
| MPNST-724 | DMEM + 10% FBS + 2 mM L-glutamine + 1 mM sodium pyruvate + P/S |
| NF90.8 | DMEM + 10% FBS + 2 mM L-glutamine + 1 mM sodium pyruvate + 10 ng/mL b-FGF + P/S |
| JH-2-002 CL | RPMI + 20% FBS + P/S |
| MPNST-642 | F12/DMEM + 10% FBS + P/S |
| sNF96.2 | DMEM + 20% FBS + P/S |
| iHSCs | DMEM + 10% FBS + P/S |

**Supplementary Figure 1. PRC2 restoration in S462-TY “addback” cell line.** Western blot showing restoration of SUZ12 and H3K27me3 with treatment of doxycycline at 500 ng/mL for 7 days.

**Supplementary Figure 2.** **PRC2 status does not alter DNA methylation patterns across global genomic features in iHSCs.** Average DNA methylation percentages quantified across specific genomic features. Data show representative replicates for each cell line (n=2 PRC2-proficient cell lines, blue; n=4 PRC2-deficient cell lines, magenta).

**Supplementary Figure 3. Differential apoptosis induction of decitabine and azacitidine.** Percent of live and apoptotic cells via flow cytometry of Annexin V and Caspase 3/7 on cells treated with decitabine, azacitidine, and DMSO control (N = 3). Cells were treated with azacitidine at its IC50 for each cell line, decitabine was treated at 0.15 μM to achieve approximately the same level of demethylation, and etoposide was treated at 20 μM for 24 h.

**Supplementary Figure 4. Decitabine reduces angiogenesis and macrophage infiltration.** Staining of CD11b, F4/80, and CD31 in tumors treated with azacitidine, decitabine, or vehicle control. Dotted line is average % tissue stained in secondary only control. Scale bar 200 um.

**Supplementary Figure 5. Azacitidine and decitabine induce the expression of many genes.** Volcano plots showing many upregulated genes in both azacitidine (left) and decitabine (right) when compared to DMSO control.
